# The Effect of U.S. University Students' Problematic Internet Use on Family Relationships: A Mixed-Methods Investigation

**DOI:** 10.1371/journal.pone.0144005

**Published:** 2015-12-11

**Authors:** Susan M. Snyder, Wen Li, Jennifer E. O’Brien, Matthew O. Howard

**Affiliations:** 1 School of Social Work, Georgia State University, Atlanta, Georgia, United States of America; 2 School of Social Work, University of North Carolina at Chapel Hill, Chapel Hill, North Carolina, United States of America; Manchester University, UNITED KINGDOM

## Abstract

This is the first study to investigate how college students in the U.S. with problematic Internet use perceive the role the Internet plays within their families of origin. The sample included 27 U.S. university students who self-identified as excessive Internet users. Participants reported spending more than 25 hours a week on the Internet on non-school or non-work-related activities and reported Internet-associated health and/or psychosocial problems. This study provides descriptive statistics from participants' completion of two problematic Internet use measures (i.e., Young's Diagnostic Questionnaire and the Compulsive Internet Use Scale) and reports findings from four focus groups. Three themes emerged from the focus groups: (1) family connectedness, (2) family conflict/family disconnection, and (3) family Internet overuse. The findings of this study are a first step toward the design of effective interventions for problematic Internet use among U.S. college students and serve to inform clinical practice and health policy in this area.

## Introduction

Studies have yet to investigate how U.S. college students with Problematic Internet Use (PIU) describe the role the Internet plays in their family relationships. It is important to examine the nexus between Internet use and family relationships because family cohesion and parental involvement can protect against addictive behaviors [1]. Studies of Internet use in the general population have found mixed results regarding the effect of the Internet on family relationships. Some scholars view Internet use as an asocial activity that detracts from opportunities for families to connect [2], whereas other scholars suggest that the Internet may create positive opportunities for families to interact [3, 4].

### PIU Defined

PIU is considered a behavioral addiction with characteristics similar to substance use disorders, and young adults have especially high risks of behavioral addictions [5]. PIU is defined as "use of the Internet that creates psychological, social, school, and/or work difficulties in a person's life" [6]. Although nearly 100% of undergraduate and graduate students use the Internet [7], estimates of current PIU using the DSM-IV-TR criteria for substance use range from 9.8% to 15.2% [8].

PIU can significantly impair daily life functioning. PIU has been linked with negative mental health consequences such as depression, attention deficit/hyperactivity disorder (ADHD), hostility, social phobia [9], problematic alcohol use [10, 11], self-injurious behavior [12], and sleep difficulties, including insomnia, snoring, sleep apnea, nightmares and difficulty staying awake during the daytime [13]. In addition, PIU has been associated with the following academic problems: absences, poor grades, and academic dismissal [14].

College students may be especially vulnerable to developing PIU because schools often provide free, unlimited Internet access [15]. Furthermore, college students typically have large blocks of unstructured time; they experience the new freedom of being away from parental control and monitoring; they may feel isolated and intimidated in a large campus community; and colleges require students to use the Internet to access course materials and to interact with professors [16].

### Internet and Family Closeness

Proponents of the view that the Internet strengthens family connections assert that people more readily share their thoughts, pictures, achievements, and daily life experiences through the Internet [17, 18]. Nearly a quarter of participants in the Pew Research Project's study of Internet and cell phone use reported that the Internet greatly improved connections with family members [19]. Williams and Merten's [20] study used data from the Pew Internet and American Life Project 2008 Networked Families Survey and found that parents reported the following improvements due to Internet: 26.8% noted increased family closeness, 75.3% endorsed increased family connection, and 54.9% believed the Internet increased communication quality. Mesch's [21] study of Internet use among Israeli adolescents found that the amount of time adolescents spent on the Internet did not diminish the time youth spent with their parents.

However, some of these studies that highlighted how the Internet strengthened family connections also shed light on how Internet use may be problematic. Mesch [21] found that adolescents who reported more frequent Internet use for non-learning purposes evidenced less familial closeness. Likewise, Williams and Merten [20] found that frequent Internet use or the presence of more devices with access to the Internet in the home decreased the amount of time families spent together, and how close they felt.

### Internet and Family Conflict

Within the family context Internet use can be a source of conflict. Conflict can emerge, for example, because the Internet creates a disconnection between physical presence and psychological presence. Thus, even when some college students are physically present with their families they may not be psychologically present as the result of their Internet use. This shift in how the family functions as a social system is a departure from homeostasis that often requires families to reconsider rules regarding communication [22–24].

Another study by Mesch [25] found that more than 40% of parents reported having conflicts with their children regarding Internet use. In Mesch's [21] earlier study he found that as adolescents used the Internet for non-academic activities, such as socializing with friends, the time they spent with their family was reduced and the amount of conflict the adolescents had with their parents increased. Family conflicts may be particularly likely among college students with PIU because the nature of PIU means they devote much of their time to online activities instead of their families. Furthermore, familial conflicts can also negatively reinforce Internet use. In other words, college students who are having conflicts with their families over their Internet use may be more likely to increase Internet use following after each conflict. It may be that the Internet serves as an escape from the negative feelings college students experience after an argument [1], or it could be that college students use social media to vent about the conflicts with their parents [26].

### Family Internet Overuse

In addition, it is important to understand whether other members of the family have PIU. Parental or sibling modeling may contribute to PIU. College students with PIU may have observed others using the Internet for meeting people, being entertained, and solving problems [15]. Although studies have not explored family history or genetic risk for Internet addiction, studies of other behavioral and substance-related addictions, such as gambling [27], and kleptomania [28], suggest that they often run in families. Thus, it would not be surprising if PIU among other family members contributes to PIU among college students, or vice versa.

### Current Study

The purpose of this mixed methods study is to increase understanding of how U.S. college students with PIU perceive the role the Internet plays within their families of origin. It is also an opportunity to understand the extent to which PIU affects daily functioning among U.S. college students. The findings of this study are a first step toward the design of effective interventions that can address PIU among U.S. college students, and serve to inform clinical practice and health policy in this area.

## Materials and Methods

This study used four focus groups with 27 participants to better understand how PIU affects college students. Between March and April, 2012 participants were recruited and assigned to one of four focus groups as their schedules permitted. Each hour-long focus group contained 6–8 participants. Prior to the start of each focus group descriptive data (i.e., sociodemographic characteristics and Internet usage) were collected.

### Focus Groups

During focus groups a facilitator guides participants with shared experiences or knowledge to share their perspectives on a specific topic [29]. The focus group method was used because 1) the target population, college students who self-identify as Internet over-users, could share their personal experiences regarding PIU; and 2) the data generated from focus groups is typically rich because group discussion can inspire participants to share personal experiences and perspectives in a manner that highlights the complexities and tensions of the topic [30].

#### Focus Group Materials and Measures

Focus group discussion was semi-structured, with the facilitator asking 22 open-ended questions (Document A in S1 File). The group discussion guide was developed and refined by the investigators based on relevant substantive theories and pilot testing. The focus groups included questions on the following topics: a) Internet use, including online activities that participants devoted most of their time to, the reasons these activities were most enjoyable, average number of hours spent daily on Internet use, and the duration of the longest continuous session of use; b) triggers that affect excessive Internet use; c) negative consequences of PIU; and d) PIU among family members. Before initiating the focus groups we pilot tested interview questions by conducting in-depth individual interviews with six university students.

The study used Young's Diagnostic Questionnaire (YDQ) [31] and Compulsive Internet Use Scale (CIUS) measures to validate participants’ self-report of PIU. Using two standardized measures triangulates the data and strengthens the validity of the results [32, 33].

The YDQ is an eight item questionnaire that evaluates preoccupation, salience, tolerance, withdrawal symptoms, and impairment of psychosocial functioning resulting from Internet use. Participants answering "yes" to 5 or more of the 8 questions were identified as having Internet addiction whereas those meeting 3 or 4 criteria were considered to have "potential Internet addiction" [31, 34]. The YDQ has been widely used in the existing literature to explore the prevalence and correlates of PIU and internet addiction among youth and young adults [35]. Thus, the results of this study can be compared to the findings of extant literature. The internal consistency reliability of the YDQ in this study was .69.

The CIUS, which assesses compulsive or addictive Internet use behavior, including losing control, being preoccupied, salience, conflict, withdrawal symptoms, and Internet use to cope with problems and dysphoric moods, includes 14 items scored with a 5-point Likert-type scale, ranging from 0 (never) to 4 (very often). Higher scores indicate greater severity of compulsive Internet use. In this study, the CIUS had an internal consistency reliability of α = .92. Although the reliability and validity of the CIUS have been established, there are currently no well validated cutoffs for the scale. Thus, this study does not make diagnoses of Internet addiction.

### Ethics Statement

The University of North Carolina-Chapel Hill Institutional Review Board approved this study in accordance with the Declaration of Helsinki. All participants provided written consent prior to the start of each focus group.

### Sample

The study used a purposive sampling approach to recruit undergraduate and graduate students, who were enrolled at a large public university in the southeastern United States. The intent of this strategy was to yield rich data regarding Internet use among students who self-identify as heavy Internet users, to determine factors that trigger PIU, and to investigate health and psychosocial consequences of PIU.

On a Friday evening the second author distributed a recruitment email to all undergraduate and graduate students who agreed to receive emails from the university student listserv. The email explained the purpose of the study, requirements for study participation, and described the research team as researchers from the School of Social Work. To be eligible for study participation individuals had to be current graduate or undergraduate students enrolled at the university, who reported intensive Internet use and problems from their Internet use. These individuals spend over 25 hours per week on the Internet for non-school or non-work-related purposes, and experience one or more physical and/or psychosocial problems as the result of intensive Internet use. To elicit a wide variety of Internet use experiences, physical and psychosocial problems were assigned a very low threshold for inclusion.

Within two hours of soliciting the study over 30 students responded to the email and expressed their interest in participating in the study; 39 students agreed to participate in focus groups. Many of the respondents indicated that their Internet use exceeded 40 hours a week for non-school and/or non-work-related reasons. These individuals also reported experiencing multiple physical and psychosocial problems due to their Internet use. Each of the 39 students who responded received an email to schedule a focus group time, and a second email to confirm that time. For reasons that are not known 12 students did not show up to the focus groups. Table 1 provides the study's sample characteristics. Participants' average age was 21 (SD = 3.6); ages ranged from 18 to 36 years old. Among the 27 study students, 63.0% (n = 17) were women; 26.0% (n = 7) identified themselves as white, 33.3% (n = 9) were black, and 33.3% (n = 9) were Asian (including one student from India), and the remaining 7.4% (n = 2) participants identified as Latino. Most participants (72.5%, n = 20) reported being undergraduates with 11 different majors.

**Table 1:** Sample Characteristics of 27 University Students Who Self-Report PIU.

	*N*	*%*	*Mean*	*S*.*D*.
Age	27	100	21	(3.6)
Gender				
Female	17	63		
Male	10	37		
Race/Ethnicity				
Asian	9	33.3		
Black	9	33.3		
Latina/Latino	2	7.4		
White	7	25.9		
Student status				
Undergraduate	22	81.5		
Graduate	5	18.5		
Major				
Biology	2	7.4		
Business/ Economics	3	11.1		
Information Science	1	3.7		
Communication/Journalism	5	18.5		
Mathematics	1	3.7		
Medicine	2	7.4		
Nutrition	1	3.7		
Philosophy	1	3.7		
Political science	3	11.1		
Psychology	2	7.4		
Public policy	2	7.4		
Sociology	2	7.4		
Undecided	2	7.4		

### Data Collection

Four focus groups lasting approximately an hour were conducted in a conference room on campus. Each group had 6 to 8 participants, which allowed for a variety of ideas and opinions to be expressed. The last author facilitated all groups, the second author attended each group and took notes, which supplemented transcription. The notes captured nonverbal communication, including body language, facial expression and tone of voice. Including more than one observer facilitated observer triangulation, which increases the reliability and validity of focus group findings [36]. Before the start of each focus group, participants completed the YDQ, the CIUS, and a short sociodemographic questionnaire. Throughout the focus groups participants answered questions about their Internet use and its effects, including how the Internet affects familial relationships.

### Data Analysis

The audio-recorded focus group sessions were transcribed verbatim by the first and second authors, and checked for accuracy by all authors. The first three authors organized codes into umbrella codes and subcodes (i.e., a code tree). Using research findings regarding correlates and consequences of PIU, codes were developed. Next, the theory-driven codes were reviewed and revised to generate codes with labels and definitions that reflected the raw data. Based on DeCuir-Gunby et al.'s [37] recommendations, during the second round of coding codes were developed on the sentence and the paragraph levels applying a data-driven approach. In this round of coding, any new themes that emerged from the data that had not been captured by the theory-driven codes were investigated and identified.

To improve the reliability and validity of the findings analytical triangulation was employed. Specifically, each author independently reviewed and coded the focus group transcriptions using the agreed upon framework. Then, as a team, the authors resolved any coding discrepancies through mutual discussion and agreement. Emergent themes and patterns were identified and categorized together by all authors until the analysis indicated convergence and saturation. The rigor of the research was advanced by triangulating data that was similar, such as collecting YDQ and CIUS, and questionnaires regarding past use. Furthermore, the research team regularly debriefed and consulted, which helped produce codes with clear functional definitions and facilitated negative case analysis [36].

## Results

### Descriptive Results

The findings from standardized assessments indicated that between 80% and 100% of the sample met criteria for PIU. Most participants reported experiencing several symptoms of PIU, including preoccupation with the Internet; repeated unsuccessful attempts to reduce or stop their Internet use; Internet use to reduce negative moods and emotions, and increasing time spent on Internet use. Over a quarter of respondents reported having lied to others to conceal the extent of their Internet use, and a third reported having risked the loss of an important life opportunity or relationship because of their Internet use.

Table 2 provides participants’ responses to the YDQ. Nearly half of the participants (48.1%, n = 13) met clinical criteria for PIU according to the YDQ, and 40.7% (n = 11) of them met criteria for potential PIU. Almost all participants (96.3%, n = 26) reported sometimes staying online longer than originally intended, with 81.5% (n = 22) of students having experienced preoccupation (i.e., thinking about the Internet and anticipating the next period of time when the Internet is not available), 74.1% (n = 20) having experienced unsuccessful efforts to control, cut back or stop their Internet use, 63.0% (n = 17) having used the Internet as means of escape, and 55.6% (n = 15) developing increasing tolerance (i.e., used the Internet with increasing amounts of time in order to achieve satisfaction). Additionally, 44.5% (n = 12) of participants reported withdrawal symptoms (i.e., felt restless, moody, depressed, or irritable) when attempting to reduce or stop their Internet use.

**Table 2:** Number and Percentage of Participants Responding Affirmatively Each of Eight Criteria for Internet Addiction on Young’s Diagnostic Questionnaire (N = 27).

Item	N	%
1.Internet preoccupation	22	81.5
2.Time using the Internet is increasing to be satisfied	15	55.6
3.Repeated unsuccessful efforts to control, cut back, or stop Internet use	20	74.1
4.Restless, moody, depressed, or irritable when try to cut down or stop Internet use	12	44.5
5.On-line longer than originally intended	26	96.3
6.Jeopardized or risked the loss of a significant relationship, job, educational, or career opportunity because of Internet use	9	33.3
7.Lied to family members, a therapist, or others to conceal the extent of your involvement with the Internet	7	25.9
8.Escape problems or relieve a dysphoric mood (e.g., feelings of helplessness, guilt, anxiety, and depression) through the Internet	17	63.0
Total YDQ Score		
YDQ ≥ 5	13	48.1
YDQ < 5	14	51.9

Table 3 provides participants’ responses to the CIUS. The entire sample exceeded the recommended cutoff for compulsive Internet use according to the CIUS. The following negative consequences due to excessive Internet use were recorded: 63.0% (n = 17) of participants reported inadequate sleep; 44.4% (n = 12) stated they neglected school work and other daily obligations due to excessive Internet use.

**Table 3:** Responses to each of 14 items on the Compulsive Internet Use Scale (N = 26).

	%(N)					
	CIUS = 0 ^a^	CIUS = 1^a^	CIUS = 2^a^	CIUS = 3 ^a^	CIUS = 4 ^a^	Mean (S.D.)
1. Difficult to stop using the Internet	7.7 (2)	0 (0)	7.7 (2)	57.5 (15)	26.9 (7)	(1.04)
2. Continue to use the Internet despite intention to stop	3.9 (1)	0 (0)	15.4 (4)	46.2 (12)	34.6 (9)	0.93)
3. Others (e.g., parents, children, parents, and friends) say you should use the Internet less	14.8 (4)	29.6 (8)	40.7 (11)	3.7 (1)	11.1 (3)	1.14)
4. Prefer to use the Internet instead of spending time with others (e.g., partner, children, parents, and friends)?	14.8 (4)	25.9 (7)	37.0 (10)	14.8 (4)	7.4 (2)	1.13)
5. Short of sleep because of the Internet	11.1 (3)	7.4 (2)	18.5 (5)	25.9 (7)	37.0 (10)	1.35)
6. Think of the Internet, even when not online	7.4 (2)	14.8 (4)	48.2 (13)	18.5 (5)	11.1 (3)	2.11 (1.05)
7. Look forward to next Internet session	7.4 (2)	22.2 (6)	37.0 (10)	18.5 (5)	14.8 (4)	1.15)
8. Think Internet use should be less often	3.7 (1)	3.7 (1)	11.1 (3)	48.2 (13)	33.3 (9)	3.03 (0.98)
9. Unsuccessfully tried to spend less time on the Internet	3.7 (1)	14.8 (4)	33.3 (9)	37.0 (10)	11.1 (3)	1.00)
10. Rush through homework to go on the Internet	15.4 (4)	19.2 (5)	26.9 (7)	30.8 (8)	7.7 (2)	1.96 (1.22)
11. Neglect daily obligations (work, school, or family life) to go on the Internet	7.4 (2)	18.5 (5)	29.6 (8)	25.9 (7)	18.5 (5)	2.30 (1.20)
12. Use the Internet when feeling down	7.7 (2)	7.7 (2)	34.6 (9)	19.2 (5)	30.8 (8)	2.58 (1.24)
13. Use the Internet to escape sorrows or get relief from negative feelings	7.7 (2)	23.1 (6)	26.9 (7)	19.2 (5)	23.1 (6)	2.27 (1.28)
14. Feel restless, frustrated, or irritated when the Internet is unavailable	14.8 (4)	11.1 (3)	29.6 (8)	29.6 (8)	29.6 (4)	2.19 (1.27)
CIUS Total Score			100 (26)			33.3 (11.2)
CIUS > 21						

a. CIUS = 0 refers to Never, CIUS = 1 refers to Seldom, CIUS = 2 refers to Sometimes, CIUS = 3 refers to Often, CIUS = 4 refers to Very often. CIUS > 21 indicates compulsive Internet use.

### Qualitative Results

Three overarching themes emerged from the focus groups relating to: a) family connectedness, b) family conflict/family disconnection and c) family Internet overuse.

#### Theme 1: Family Connectedness

This theme describes the ways the Internet brings families together. Many participants described how the Internet positively impacts family relationships. Subthemes included: a) connecting with extended family and b) connecting with nuclear family.

For some participants the Internet connected them with extended family. "Janet" shared how the Internet connected her to extended family that she had not previously met, "Um, for me, like, I get connected with family who I've never seen or known them very well."

Other participants described how the Internet allows them to stay connected to their family of origin. For some students the Internet helped them stay connected to their parents when they went to college out of state. "Lisa" and "Tiffany" both shared that they did not like using the phone, so the Internet facilitates communication that would not otherwise have happened, as "Lisa" explained:

I hate talking on the phone. So, that allows me a way to stay connected and especially with my mom who would…Normally, I would just not respond to her at all, but now we have an email dialogue going. That helps us stay more connected.

Participants described how using Skype allows them to see things as if they were at home. "Hannah" explained:

But like using Skype helps keep you connected and also when we are at home we watch a movie together, it's like family time, you know. And um, like you know, if we read the same, like article, then we can talk about it on Skype.

Participants seemed to agree that the Internet allows them to exchange ideas or talk about things they have seen online. For example, "Carla" explained that she and her mother are regularly "shooting each other articles or funny links on Facebook."

#### Theme 2: Family Conflict/Family Disconnection

Not all experiences with the Internet brought families of college students with PIU closer together. Some participants described how their Internet use sparked family conflict. Subthemes included: a) disagreements over content posted, and b) disconnecting from family.

Some of the conflict concerned the content of what had been posted online on social media sites like Facebook. "Stephanie" shared,

There have been a few times that my parents are like 'Hey, we saw something that we don't like,' or 'Like your uncle, who's your Facebook friend, told me that he saw this picture,' and it has caused a lot of unnecessary drama. Just maybe it's like he said, she said, he said, or whatever justification. My parents are pretty strict though, so they don't really understand.

For college students with PIU the Internet can disconnect them from opportunities to engage with their family. Most participants shared their experiences of becoming disconnected from their families while online. "Steve" shared an experience that occurred when his brother went to a sports bar with the participant and his friends,

At one point we're all watching the basketball game, and all four because we're all on our phone, and he looked at us and he said, 'Really guys, I am here for two days, you all just wanna [*sic*] be on Twitter and Facebook?' So, while it can enhance with setting up social situations, it can also detract from them once you were actually in them…Yeah, he was very just like…He flew out for the weekend. You know he spent $300 on an airplane ticket just to sit there and watch me on Facebook.

Other participants were cognizant of the fact that their time at home was more focused on the Internet than family interactions. Several participants said that when they went home they were "on the computer the whole time." There was also consensus that parents did not understand why participants spend so much of their time on "Twitter," "Facebook," and other social media. "Michael" explained that his mother was not happy about how absorbed he got into social networking videos, whereas another explained she would largely ignore her family when she was at home because of her Internet use.

My grandma, and my parents will complain about my Internet use because I will be sitting in front of the TV and I'll have my laptop and so will my little sister. We'll be sitting in front of the TV on our laptops not talking to each other. So, my parents will complain about that.

Some participants would misinterpret when a conversation was over and would go online at times that created conflict, such as during family meals. As "Sarah" described,

My mom talks about me using the phone at the table when we're eating, cause like if there's a break in conversation, 'Oh, Facebook opportunity' [others laughed and she laughed too]. And then, like, somehow in my mind conversation is over, but it's really not. So then she's like 'You're always on your phone, what are you doing?' But then, like two minutes later, she is checking the weather. So I don't know [she laughed].

#### Theme 3: Family Internet Overuse

This theme refers to Internet overuse among other family members. When the facilitator asked participants to identify other family members with PIU, study participants provided several examples, including siblings, parents and cousins. Subthemes that emerged were (1) the need for parental limits; (2) gaming addictions; (3) parental Internet use for work; and, (4) parents who do not use the Internet.

On the theme of the need for parental limits, several participants expressed concern about the extent to which younger siblings or other family members used the Internet. From their perspective their family boundaries became more permeable because parents were not enforcing rules that the participants felt should exist. As "Melissa" explained about her little brother,

He just turned four, but they got him an iPad. Like, which I don't agree with. I think it's so stupid, but he is always, always on it. He gets really defensive if you try to take it away or put boundaries on it or something like that.

Another participant, "Michelle" shared her concern about her sister's Internet use, and her parents' lack of awareness regarding her sister's use.

Quite a few times my parents have called me, and they like think she's in bed because they are asleep and then they will wake up. She'll be like IMing someone in the middle of the night, like she has these friends in Paris, they'll be video chatting—it's not a normal hour or time.

Several participants shared that they had a family member whose Internet gaming was problematic. Each instance of problematic gaming involved male family members. As "Stephanie" explained,

My older brother, he is 24. He just graduated from college. He is at home, and trying to get a job. He is always on the computer and my parents are like, 'Get out of the house.' When he is at the computer he is playing Xbox Live, which is on the Internet too. I think he is addicted to it, but… [she laughed].

"Hannah" explained that even though her cousin's Internet gaming has impaired his vision he is unable to stop:

My cousin, he is addicted to video games. And he's like, I think he is like 10, 12, something like that, I don't remember. I feel like it's a stupid game, there's no deepness to it. You kill someone. They die. You get killed, it starts over again. He can play that for 8 hours straight without moving. His eyes are really bad right now. He can't control himself.

Several participants stated that their parents were on the Internet all the time. Participants whose parents used the Internet for work described their parents as "constantly checking email." Others noted that their parents were regularly on computers, phones, or iPads "on Facebook" or "browsing."

While, most participants said that their sister, dad, or mother were addicted to the Internet, a few participants shared that no one else in the family engaged in PIU. "Cindy" said that the rest of her family grew up in another country, which may explain why they do not use the Internet, "I find that I don't really have family members with an Internet problem, and I am the only one who grew up here. So, that might be…" Another participant, "Gina" said, "My parents are technophobes. They don't even know how to turn on computers."

## Discussion

This study's results extend understanding of how college students with PIU describe the role of the Internet within their families. The findings from the focus groups highlighted the complicated role the Internet plays in both bringing families together and disconnecting families. These findings also conveyed that for most of the participants PIU was experienced by other members of the family. Thus, it appears that students suffering from PIU are able to accurately identify and report these problems, many of which are severe.

Consistent with Wilcox and Stephen [17], even among a group of students who experienced PIU, we found that many participants reported that the Internet allowed them more easily to connect with their families and share daily life experiences. The focus groups also supported William and Merten's [20] study by providing rich evidence of the feelings of closeness and connection among families. Participants explained how the Internet facilitated connections with family through email, social media, and Skype. These participants described the Internet as a preferred medium for exchanging ideas or talking about things. Similar to Mesch [21] we found that while college students with PIU did spend time with their families, most reported that the time they spent online prevented them from fully connecting with their family during visits. However, none of the participants in our focus groups explicitly stated that they felt less close to their family as the result of their Internet usage.

While some participants reported that the Internet brought them closer to their families, others reported that the Internet disconnected them from their families. These findings were similar to the findings of Shek et al.'s [1] review of the literature. In particular, our study participants explained that when they went home they spent more of their time online than interacting with their families. While we established that college students' PIU detracted from family time, it would be helpful if future studies would delve more deeply into the issue of family cohesion to further our understanding of how a lack of family cohesion triggers the genesis of PIU.

Like Mesch [24], we found that most participants reported having conflict with members of their family over their Internet use. Some participants explained that things they had posted online upset their parents and other relatives. Most participants also shared experiences where their use upset their parents or other family members because they were not engaging with their families while at home. This finding is consistent from Li et al.'s [35] systematic review on family factors in Internet addiction among Chinese youth. Youth with PIU reported higher level of child-parent conflict than youth without PIU.

One of the most interesting findings was the extent to which participants shared that other members of their families engaged in PIU as well. Some participants were especially concerned about PIU among their younger siblings, and expressed a desire for their parents to set limits. It is unclear to what extent PIU was modeled within the family or may be due to shared genetic factors. It is possible that the college students in our study have inadvertently modeled PIU for their younger siblings.

This study has some limitations that future studies should address. The small sample size, gender imbalance, and limited geographic scope of the study are limitations. However, the study [24] also had a number of strengths. The university where this research was conducted is similar to other large public universities in demographic composition. Further, the data included quantitative and qualitative assessments. Perhaps most importantly, given the dearth of research on U.S. university students with PIU, our results have the potential to encourage further investigation in this emerging area.

PIU among college students is a complex problem that is not easily addressed. However, additional steps can be taken in the areas of research and practice.

Beginning with research, future studies of PIU should build on the results of this exploratory study. In particular it would be helpful to explore the extent to which families experience conflict or become disconnected as a result of college students' PIU, using large nationally representative samples of college students in the U.S. Likewise, it would be informative to follow individuals with PIU to determine how PIU evolves over time. It would also be interesting if qualitative researchers were to interview the parents and siblings of college students with PIU to better understand how PIU has affected the family.

With regard to practice, when working with college students who have PIU it is important to examine the extent to which Internet use has affected physical and emotional health. It is also a good idea to assess whether the student's Internet use has strained relationships with family members or friends. In particular, it would be useful to assess whether the family has become disconnected due to the college student's Internet use, or if familial conflicts have surrounded the college student's PIU. At the same time it is important to explore how pervasive PIU is within the family context. If a college student seeking treatment has parents and siblings who also have PIU this could be valuable information for understanding potential barriers to treatment.

## Supporting Information

S1 File (DOCX)
