# Supplementary material for: The Effect of U.S. University Students' Problematic Internet Use on Family Relationships: A Mixed-Methods Investigation
Source: PLoS One. 2015 Dec 11;10(12):e0144005. doi: 10.1371/journal.pone.0144005 (PMC4676703; doi:10.1371/journal.pone.0144005)
Supplement: S1 File — (DOCX) [file pone.0144005.s001.docx]

**Survey A**. Survey Questions for Sociodemographic and IA/PIU Characteristics, and Focus Group Discussion Guidance

**I. Sociodemographic Information and Survey Questions**

- **Sociodemographic information**

1. How old are you (in years)?
2. How would you describe your race or ethnicity (African American, White, Latino/Latina, Asian, Native American, Biracial, Other)?
3. How would you describe your gender (male, female, transgendered)?
4. Are you an undergraduate or graduate student?
5. What is your major (if undergraduate) or area of study (if graduate student)?

- **Young’s Diagnostic Questionnaire**

1. Do you feel preoccupied with the Internet (think about previous online activity or anticipate next online session)?

YES NO

1. Do you feel the need to use the Internet with increasing amounts of time in order to achieve satisfaction?

YES NO

1. Have you repeatedly made unsuccessful efforts to control, cut back, or stop Internet use?

YES NO

1. Do you feel restless, moody, depressed, or irritable when attempting to cut down or stop Internet use?

YES NO

1. Do you stay on-line longer than originally intended?

YES NO

1. Have you jeopardized or risked the loss of a significant relationship, job, educational, or career opportunity because of the Internet?

YES NO

1. Have you lied to family members, a therapist, or others to conceal the extent of your involvement with the Internet?

YES NO

1. Do you use the Internet as a way of escaping from problems or of relieving a dysphoric mood (e.g., feelings of helplessness, guilt, anxiety, and depression)?

YES NO

- **The Compulsive Internet Use Scale**

The following questions should be answered about your use of the Internet for private purposes. Answers can be given on a 5-point scale: (0) Never, (1) Seldom, (2) Sometimes, (3) Often, and (4) Very often.

1. How often do you find it difficult to stop using the Internet when you are online?
2. How often do you continue to use the Internet despite your intention to stop?
3. How often do others (e.g., partner, children, parents, and friends) say you should use the Internet less?
4. How often do you prefer to use the Internet instead of spending time with others (e.g., partner, children, parents, and friends)?
5. How often are you short of sleep because of the Internet?
6. How often do you think of the Internet, even when not online?
7. How often do you look forward to your next Internet session?
8. How often do you think you should use the Internet less often?
9. How often have you unsuccessfully tried to spend less time on the Internet?
10. How often do you rush through your homework in order to go on the Internet?
11. How often do you neglect your daily obligations (work, school, or family life) because you prefer to go on the Internet?
12. How often do you go on the Internet when you are feeling down?
13. How often do you use the Internet to escape from your sorrows or get relief from negative feelings?
14. How often do you feel restless, frustrated, or irritated when you cannot use the Internet?

- **The experience of problem Internet use**

1. How old were you when you first started to use the Internet?
2. How old were you when you first thought you might have a problem with overuse of the Internet?
3. Do you consider your Internet problem very mild, mild, moderate or severe?
4. If an effective treatment for Internet overuse were available, would you be interested in participating in it?

**II. Questions for focus group discussion**

1. Which online activities do you spend the most time on (e.g., playing on-line games, browsing, updating personal social webpage, and chatting with others in chatrooms or via instant messenger)?
2. Why do you enjoy this (these) online activity (ies) the most? What is it about the Internet that you enjoy?
3. How much time, on average, do you spend on the Internet per day?
4. Generally, what is the longest period of time you have ever spent on the Internet in one continuous session of use?
5. What emotional, interpersonal, or situational factors trigger your use of the Internet (heighten your desire to use)? What factors do you think played the strongest role in contributing to the development of your Internet overuse problem?
6. How many of your friends have problems with Internet overuse? If yes, what kind of problems do they have?
7. How many of your family members have problems with Internet overuse? If yes, what kind of problems do they have?
8. Do you consider your Internet problem very mild, mild, moderate or severe? Why?
9. Have you made efforts to control, cut back, or reduce the time of your Internet use? Did you succeed? How did you do it?
10. What is the longest period of time you have gone without getting on the Internet in the past year? How did you feel during this period emotionally and was your functioning affected in any way? How did you cope with it?
11. What would be the hardest aspect of significantly reducing the time you spend on the Internet?
12. Does excessive Internet use have any kind of influence on your physical health? For example, does the Internet use disturb your sleep pattern in some way? Do you feel fatigued generally because of your excessive Internet use? Is there anything else?
13. Does excessive Internet use or any specific online activities have any kind of influence on your mood or feelings? Do these online activities help you to relieve negative feelings (e.g., anxiety, loneliness, depression)? Please explain.
14. Does your Internet use have any kind of influence on your social life? Does the Internet replace some of the interpersonal interaction in your daily life, such as going out to meet friends, and going out to meet new people? If yes, how?
15. Does your Internet use have any kind of influence on your school or work?
16. Has your relationship with your family suffered as a result of your Internet overuse? If so, in what ways?
17. What do people around your say about your Internet use? Do people around you, such as parents, roommates, classmates, friends, etc. complain about your Internet use? If yes, what do they say? How do you feel about their complaints?
18. Do you use alcohol or other drugs when using the Internet? Do you see any relationships between your substance use and your Internet use?
19. What consequences of your Internet overuse bother you the most?
20. How is your Internet overuse decreasing the quality of your life currently?
21. If an effective treatment for Internet overuse were available, would you be interested in participating in it?
22. Do you have anything else to add about the causes or consequences of your Internet over use that will help us to understand Internet over use behavior?

| **Table A** Number and percentage of participants responding affirmatively each of eight criteria for Internet Addiction on Young’s Diagnostic Questionnaire (N = 27) | | | |
| --- | --- | --- | --- |
| Item |  | % (N) |  |
| 1. Internet preoccupation |  | 81.5% (22) |  |
| 1. Time using the Internet is increasing to be satisfied |  | 55.6% (15) |  |
| 1. Repeated unsuccessful efforts to control, cut back, or stop Internet use |  | 74.1% (20) |  |
| 1. Restless, moody, depressed, or irritable when try to cut down or stop Internet use |  | 44.5% (12) |  |
| 1. On-line longer than originally intended |  | 96.3% (26) |  |
| 1. Jeopardized or risked the loss of a significant relationship, job, educational, or career opportunity because of Internet use |  | 33.3% (9) |  |
| 1. Lied to family members, a therapist, or others to conceal the extent of your involvement with the Internet |  | 25.9% (7) |  |
| 1. Escape problems or relieve a dysphoric mood (e.g., feelings of helplessness, guilt, anxiety, and depression) through the Internet |  | 63.0% (17) |  |
| Total YDQ Score |  |  |  |
| YDQ > 5 |  | 48.1% (13) |  |
| YDQ < 5 |  | 51.9% (14) |  |
| YDQ > 5 indicates Internet addiction. YDQ scores of 3 or 4 = potential IA. | | | |

**Table B** Data Set for Young’s Diagnostic Questionnaire (N = 27)

| Case ID | YDQ Score | Item1 | Item2 | Item3 | Item4 | Item5 | Item6 | Item7 | Item8 |
| --- | --- | --- | --- | --- | --- | --- | --- | --- | --- |
| 1 | 4 | 1 | 1 | 1 | 0 | 1 | 0 | 0 | 0 |
| 2 | 5 | 1 | 0 | 1 | 0 | 1 | 1 | 0 | 1 |
| 3 | 6 | 1 | 1 | 1 | 1 | 1 | 0 | 0 | 1 |
| 4 | 4 | 1 | 1 | 1 | 0 | 1 | 0 | 0 | 0 |
| 5 | 7 | 1 | 1 | 1 | 1 | 1 | 0 | 1 | 1 |
| 6 | 4 | 1 | 0 | 1 | 0 | 1 | 0 | 0 | 1 |
| 7 | 7 | 1 | 1 | 1 | 1 | 1 | 0 | 1 | 1 |
| 8 | 8 | 1 | 1 | 1 | 1 | 1 | 1 | 1 | 1 |
| 9 | 6 | 1 | 1 | 1 | 0 | 1 | 1 | 0 | 1 |
| 10 | 5 | 1 | 0 | 1 | 1 | 1 | 1 | 0 | 0 |
| 11 | 8 | 1 | 1 | 1 | 1 | 1 | 1 | 1 | 1 |
| 12 | 6 | 1 | 0 | 1 | 1 | 1 | 1 | 0 | 1 |
| 13 | 4 | 0 | 0 | 1 | 1 | 1 | 0 | 0 | 1 |
| 14 | 5 | 1 | 1 | 0 | 0 | 1 | 0 | 1 | 1 |
| 15 | 4 | 1 | 1 | 1 | 0 | 1 | 0 | 0 | 0 |
| 16 | 0 | 0 | 0 | 0 | 0 | 0 | 0 | 0 | 0 |
| 17 | 3 | 0 | 0 | 1 | 0 | 1 | 0 | 0 | 1 |
| 18 | 2 | 0 | 0 | 1 | 0 | 1 | 0 | 0 | 0 |
| 19 | 2 | 1 | 0 | 0 | 0 | 1 | 0 | 0 | 0 |
| 20 | 4 | 1 | 1 | 0 | 0 | 1 | 0 | 1 | 0 |
| 21 | 4 | 0 | 1 | 1 | 0 | 1 | 1 | 0 | 0 |
| 22 | 4 | 1 | 0 | 0 | 1 | 1 | 0 | 0 | 1 |
| 23 | 7 | 1 | 1 | 1 | 1 | 1 | 1 | 1 | 0 |
| 24 | 7 | 1 | 1 | 1 | 1 | 1 | 1 | 0 | 1 |
| 25 | 3 | 1 | 0 | 0 | 0 | 1 | 0 | 0 | 1 |
| 26 | 6 | 1 | 1 | 1 | 1 | 1 | 0 | 0 | 1 |
| 27 | 3 | 1 | 0 | 0 | 0 | 1 | 0 | 0 | 1 |

**Table C** Data set for the Compulsive Internet Use Scale (N = 27)

| Case ID | CIUS Score | Item1 | Item2 | Item3 | Item4 | Item5 | Item6 | Item7 | Item8 | Item9 | Item10 | Item11 | Item12 | Item13 | Item14 |
| --- | --- | --- | --- | --- | --- | --- | --- | --- | --- | --- | --- | --- | --- | --- | --- |
| 1 | 32 | 3 | 2 | 1 | 1 | 3 | 3 | 3 | 2 | 2 | 3 | 3 | 2 | 1 | 3 |
| 2 | 36 | 3 | 3 | 0 | 2 | 4 | 2 | 1 | 3 | 3 | 3 | 2 | 4 | 4 | 2 |
| 3 | 36 | 3 | 3 | 2 | 1 | 3 | 2 | 1 | 4 | 4 | 2 | 3 | 3 | 2 | 3 |
| 4 | 32 | 3 | 3 | 2 | 2 | 2 | 2 | 2 | 4 | 3 | 2 | 2 | 2 | 1 | 2 |
| 5 | 51 | 4 | 4 | 2 | 4 | 4 | 3 | 4 | 3 | 3 | 4 | 4 | 4 | 4 | 4 |
| 6 | 29 | 3 | 3 | 1 | 1 | 1 | 2 | 3 | 4 | 2 | 3 | 2 | 2 | 2 | 0 |
| 7 | 50 | 4 | 4 | 3 | 3 | 4 | 4 | 4 | 3 | 3 | 3 | 4 | 4 | 4 | 4 |
| 8 | 54 | 4 | 4 | 4 | 4 | 4 | 4 | 4 | 4 | 4 | 4 | 4 | 3 | 3 | 4 |
| 9 | 48 | 4 | 4 | 4 | 3 | 4 | 2 | 1 | 4 | 3 | 3 | 4 | 4 | 4 | 4 |
| 10 | 34 | 3 | 3 | 2 | 2 | 4 | 2 | 2 | 4 | 3 | 1 | 3 | 2 | 1 | 2 |
| 11 | 33 | 2 | 2 | 2 | 3 | 2 | 3 | 3 | 3 | 2 | 2 | 1 | 3 | 2 | 3 |
| 12 | 35 | 3 | 4 | 2 | 1 | 2 | 2 | 2 | 3 | 3 | 1 | 3 | 3 | 3 | 3 |
| 13 | 40 | 5 | 5 | 0 | 0 | 4 | 3 | 2 | 4 | 1 | 2 | 1 | 5 | 5 | 3 |
| 14 | 30 | 3 | 3 | 2 | 1 | 1 | 2 | 2 | 2 | 2 | 3 | 3 | 2 | 2 | 2 |
| 15 | 20 | 4 | 3 | 0 | 0 | 2 | 0 | 0 | 3 | 2 | 1 | 0 | 1 | 3 | 1 |
| 16 | 0 | 0 | 0 | 0 | 0 | 0 | 0 | 0 | 0 | 0 | 0 | 0 | 0 | 0 | 0 |
| 17 | 23 | 0 | 2 | 2 | 1 | 3 | 1 | 1 | 3 | 3 | 1 | 2 | 2 | 2 | 0 |
| 18 | 17 | 3 | 3 | 1 | 0 | 0 | 2 | 1 | 4 | 2 | 0 | 1 | 0 | 0 | 0 |
| 19 | 31 | 3 | 3 | 2 | 2 | 3 | 2 | 2 | 3 | 1 | 2 | 2 | 2 | 2 | 2 |
| 20 | 28 | 3 | 3 | 1 | 2 | 3 | 2 | 2 | 3 | 2 | 1 | 1 | 2 | 1 | 2 |
| 21 | 32 | 3 | 3 | 2 | 3 | 3 | 1 | 1 | 3 | 3 | 3 | 3 | 2 | 1 | 1 |
| 22 | 34 | 3 | 3 | 1 | 2 | 4 | 2 | 3 | 1 | 1 | 2 | 2 | 4 | 4 | 2 |
| 23 | 41 | 4 | 4 | 1 | 2 | 3 | 4 | 4 | 2 | 2 | 3 | 4 | 3 | 2 | 3 |
| 24 | 43 | 4 | 4 | 2 | 2 | 2 | 3 | 3 | 3 | 4 | 2 | 3 | 4 | 4 | 3 |
| 25 | 37 | 3 | 4 | 4 | 2 | 4 | 2 | 2 | 4 | 1 | 0 | 2 | 4 | 3 | 2 |
| 26 | 28 | 3 | 2 | 1 | 2 | 0 | 1 | 2 | 3 | 3 |  | 1 | 4 | 3 | 3 |
| 27 | 25 | 2 | 4 | 1 | 1 | 4 | 1 | 2 | 3 | 2 | 0 | 2 | 1 | 1 | 1 |
